# Gut Lignocellulose Activity and Microbiota in Asian Longhorned Beetle and Their Predicted Contribution to Larval Nutrition

**DOI:** 10.3389/fmicb.2022.899865

**Published:** 2022-05-09

**Authors:** Lixiang Wang, Chunchun Li, Xuan Wang, Gaijin Wang, Suqin Shang, Zhipeng Dou, Youqing Luo

**Affiliations:** ^1^Biocontrol Engineering Laboratory of Crop Diseases and Pests of Gansu Province, College of Plant Protection, Gansu Agricultural University, Lanzhou, China; ^2^Chinese Academy of Forestry Sciences, Beijing, China; ^3^Beijing Key Laboratory for Forest Pest Control, Beijing Forestry University, Beijing, China; ^4^Sino-France Joint Laboratory for Invasive Forest Pests in Eurasia, Beijing Forestry University, Beijing, China

**Keywords:** gut microbiota, lignocellulose, gut structure, *Anoplophora glabripennis*, host tree resistance, larval nutrition

## Abstract

*Anoplophora glabripennis* (Asian longhorned beetle) is a wood-boring pest that can inhabit a wide range of healthy deciduous host trees in native and invaded areas. The gut microbiota plays important roles in the acquisition of nutrients for the growth and development of *A. glabripennis* larvae. Herein, we investigated the larval gut structure and studied the lignocellulose activity and microbial communities of the larval gut following feeding on different host trees. The larval gut was divided into foregut, midgut, and hindgut, of which the midgut is the longest, forming a single loop under itself. Microbial community composition and lignocellulose activity in larval gut extracts were correlated with host tree species. *A. glabripennis* larvae fed on the preferred host (*Populus gansuensis*) had higher lignocellulose activity and microbial diversity than larvae reared on either a secondary host (*Salix babylonica*) or a resistant host (*Populus alba* var. *pyramidalis*). *Wolbachia* was the most dominant bacteria in the gut of larvae fed on *S. babylonica* and *P. alba* var. *pyramidalis*, while *Enterococcus* and *Gibbsiella* were the most dominant in larvae fed on *P. gansuensis*, followed by *Wolbachia*. The lignocellulose-degrading fungus *Fusarium solani* was dominant in the larval gut fed on different host trees. Functional predictions of microbial communities in the larval gut fed on different resistant host trees suggested that they all play a role in degrading lignocellulose, detoxification, and fixing nitrogen, which likely contribute to the ability of these larvae to thrive in a broad range of host tree species.

## Introduction

Insect herbivores inhabit diverse habitats and feed on various substrates. Like other animals, insects are colonized by microorganisms including bacteria, fungi, protozoa, and archaea (Douglas, 2015). Insect guts connect the interior of the insect with the external environment, and they harbor diverse microbial assemblages (Dillon and Dillon, 2004; Engel and Moran, 2013). Many studies have reported complex interactions between gut microbes and host insects that are important for both organisms (Warnecke et al., 2007; Douglas, 2015; Peter and Fernando, 2020). For example, insect guts provide living space for microorganisms, and in turn, microorganisms in the gut provide support to their hosts in the form of nutrition, digestion, development, reproduction, defense, behavior, and survival (Prasad et al., 2018; Hassan and Martin, 2022). Studies have reported that gut microbiota also has negative even detrimental effects on host insects (Ge et al., 2017; Khaeso et al., 2017; Xu et al., 2019). With developments in molecular biology, research on the gut microbial community and biological functions of insects has gradually increased, and the results have potential implications for pest control (Lemoine et al., 2020).

The xylem is a nutritionally poor, recalcitrant substrate containing refractory lignocellulosic bonds (Geib et al., 2008; Brune, 2014). Wood-feeding (xylophagous) insects can adapt to a range of ecological niches, where they often thrive on the xylem of nutrient-poor or refractory species because of the contributions of gut microbes to host nutrition are particularly important in wood-boring insects (Brune and Dietrich, 2015). Gut microbes can degrade lignocellulose and release glucose and other fermentable sugars from recalcitrant plant cell wall carbohydrates, including cellulose and hemicellulose, helping wood-boring pests in terms of digestion, absorption, and utilization of lignocellulose, and providing essential nutrients such as amino acids, as demonstrated for *Saperda vestita* (Delalibera et al., 2005), *Dendroctonus valens* (Cheng et al., 2018), *Sirex noctilio* (Li et al., 2021), and others. The microbiota components that mediate these interactions consist primarily of fungi and bacteria. However, the gut microbial assemblages and functions of borers may vary considerably depending on gut morphology, insect species, and host plant (Warnecke et al., 2007; Schauer et al., 2012; Ceja-Navarro et al., 2013; Mikaelyan et al., 2017).

The Asian longhorned beetle, *Anoplophora glabripennis* Motschulsky, is a wood-feeding insect that requires international quarantine. *A. glabripennis* is highly polyphagous and found on a wide range of tree species, including species *Acer*, *Populus*, *Salix*, *Ulmus*, *Betula* and *Aesculus* (Cavey et al., 1998; Luo et al., 2003). Unlike most cerambycids that feed on stressed, dying, or dead trees, *A. glabripennis* is among the most feared invasive insect species worldwide because it attacks healthy, vigorous trees, in addition to weakened trees. Females lay eggs underneath the bark at the phloem-cambium interface. Newly hatched larvae feed subcortically along the phloem and bark, and the second instar larvae begin boring into the xylem and heartwood to feed (Luo et al., 2003). Larval feeding disrupts vascular tissue, which girdles the tree and eventually causes death.

In a nutrient-poor environment, beetle larvae utilize a number of strategies to contend with the nutritional challenges of feeding on living trees. The genome of *A. glabripennis* contains an extensive repertoire of genes involved in lignocellulose digestion and metabolism of xenobiotics (Scully et al., 2013a,b; McKenna et al., 2016). Previous research showed that lignin, cellulose, and hemicellulose degradation occur within the gut of larval *A. glabripennis* (Geib et al., 2008, Geib et al., 2009, 2010). These lignocellulolytic enzymes may originate from gut symbionts, ingestion of enzymes produced by wood decay fungi, or the insect itself (Martin, 1983; Brune, 2003; Suh et al., 2005). However, *A. glabripennis* larvae harbor a diversity of bacteria and fungi in their gut that have putative roles in nutrient provisioning, lignocellulose metabolism, and allelochemical metabolism (Geib et al., 2009; Mason et al., 2019). For example, bacteria can fix and recycle nitrogen (Scully et al., 2013a,b, 2014; Ayayee et al., 2016). A filamentous fungus (*Fusarium solani*) is consistently associated with the larval stage, and this species can degrade lignocellulose and cell wall polysaccharides and extract nutrients from woody tissues (Geib et al., 2012; Scully et al., 2013a,b; Herr et al., 2016).

In the beetle species that harbored gut microbes, a broader diversity of microbes was associated with broader tree host range. *A. glabripennis* larvae reared in a preferred host (*Acer saccharum*) had the highest gut bacterial diversity compared with larvae reared either in a resistant host (*Pyrus calleryana*) (Geib et al., 2010). An extensive investigation of the different tree species attacked by *A. glabripennis* conducted in north-western China and found that it has different host suitability. *Populus gansuensis* is the preferred host for *A. glabripennis*, followed by *Salix babylonica*, with *Populus alba* var. *pyramidalis* being more resistant. However, *A. glabripennis* was able to complete its development in all these species. Whether changed microbiota in *A. glabripennis* fed on different host trees would lead to varied microbial functions and further influence the fitness of the insect is unclear.

This study aimed to examine relationships between host tree species, gut microbial community composition, and lignocellulose digestion in *A. glabripennis*. We studied the gut structure of larvae and compared gut microbial communities and lignocellulose activities of larvae in a preferred host (*Populus gansuensis*: EBY), an alternative host (*Salix babylonica*: LS), and a third tree species (*Populus alba* var. *pyramidalis*: XJY) highly resistant to *A. glabripennis*. We also predicted the function of microbial communities in the gut of larvae feeding on different host tree species. This study may unlock novel strategies for the development of pest management approaches based on interfering with the gut microbiota and restricting their role in larval survival and development.

## Materials and Methods

### Insect Collection and Gut Dissection

New oviposition pits within a week were marked in the artificial mixed forest plantation attacked by *A. glabripennis* from the Jiuquan City, Gansu Province, Northwest China (39.71°N, 98.5°E) in October 2019. Overall, nine trees were selected (three for each of *P. gansuensis*, *S. babylonica*, and *P. alba* var. *pyramidalis*), and each sample tree was marked with fresh oviposition pit. *A. glabripennis* require 2 years to complete its life cycle in that area. All trees were collected in April 2020, at which time the larvae were at the second instar stage (boring into nutrient-poor xylem to feed). Felled trees were cut into ∼50 cm bolts and stored in barrels until transportation. Infested material was transported to the quarantine room at Gansu Agricultural University. Larvae were collected using a wood splitter (MX-3326, Qufu Mingxin Machinery Equipment Co., Ltd., Shandong, China).

Before gut dissection, we prepared sterile water, 75% (v/v) ethanol, and phosphate-buffered saline (PBS). All work areas and instruments were surface-sterilized with 75% (v/v) ethanol. After being removed from logs, *A. glabripennis* larvae were anesthetized on ice, surface-sterilized, fixed on a wax plate, and carefully dissected using a dissection microscope (Shanghai Yongke Optical Instrument Co., Ltd., Shanghai, China) under aseptic conditions (Mason et al., 2017). A pair of microscissors was used to cut larvae from the end of the abdomen to the head. The gut was gently removed and placed in sterile water to wash off any attached fat bodies or other tissues. If visible damage to the gut was observed, the insect was discarded. Guts were placed in a sterilized 1.5 mL centrifuge tube, immediately flash-frozen in liquid nitrogen, and stored at −80°C until DNA extraction. Larval guts were observed under a Leica M205FA stereoscopic microscope (Leica, United States), and different parts were analyzed in detail.

### Preparation of Enzyme Solution

Crude gut enzyme extracts were prepared from pooled samples of three larval guts. Three independent repetitions were performed for each tree species (*P. gansuensis*, *S. babylonica*, and *P. alba* var. *pyramidalis*). Gut dissections were performed as described above. Pooled gut samples were suspended in 2 mL of acetic acid-sodium acetate buffer (pH 5.2, 0.1 M) and homogenized with a micropestle on ice. Samples were centrifuged at 12,000 *g* for 20 min at 4°C and the supernatant was collected in a fresh centrifuge tube, stored at −30°C, and used as an enzyme solution to measure ligninase and cellulase activities.

### Determination of Lignocellulolytic Enzyme Activity

To determine the effect of the host tree on gut cellulolytic activity, *in vitro* activities of β-1,4-exoglucanase, β-1,4-glucosidase, and β-1,4-endoglucanase was measured in crude gut extracts incubated with cellulose substrates based on the release of reducing sugars assessed by dinitrosalicylic acid (DNS) assay (Miller, 1959). For β-exoglucanase activity, 200 μL of a 1% avicel solution was combined with 100 μL of crude gut extract (Kukor et al., 1988). For β-glucosidase activity, 200 μL of a 1% salicin solution was combined with 100 μL of diluted enzyme solution (crude gut extract diluted five times with acetic acid-sodium acetate buffer) (Li et al., 2010). For β-endoglucanase activity, 200 μL of 1% carboxymethylcellulose solution was combined with 100 μL of diluted enzyme solution (Kukor et al., 1988). After incubating at 40°C for 60 min, 300 μL of DNS reagent was added to halt enzyme activity. Samples were immediately incubated in a boiling water bath for 5 min, and absorbance was read at 540 nm on a SpectraMax microplate reader (Molecular Devices, Sunnyvale, CA, United States) along with glucose standards.

In this study, three lignin-degrading enzymes were examined in crude gut extracts. Lignin peroxidase (LiP) activity was measured based on veratryl alcohol (Pinto et al., 2012), and the change in absorbance at 310 nm was determined within 3 min of reaction. The reaction mixture (3 mL) comprised 0.1 mL of veratryl alcohol with 0.1 mL of enzyme solution, 0.1 mL of H_2_O_2_, and 2.7 mL of tartaric acid-sodium tartrate. Manganese peroxidase (MnP) activity was determined based on the oxidation rate of MnSO_4_ (Xu et al., 2017), and the change in absorbance at 270 nm was determined within 3 min. The sample mixture (4 mL) contained 0.1 mL of MnSO_4_ substrate, 0.4 mL of enzyme solution, 0.1 mL of H_2_O_2_, and 3.4 mL of tartaric acid-sodium tartrate. Laccase activity (Lac) was determined with ABTS as substrate (Kumari and Das, 2016), and the change in absorbance at 420 nm was measured within 3 min. Briefly, 0.2 mL of ABTS was mixed with 2.7 mL of tartaric acid-sodium tartrate and 0.1 mL of enzyme solution. One unit of enzyme activity (LiP, MnP, and Lac) was defined as the amount of enzyme required to produce 1 μmol of product per min at the temperature employed.

### Total DNA Extraction, PCR Amplification, and Sequencing

Total DNA was extracted from gut tissue using a ZR Fecal DNA MiniPrep Kit (Zymo Research, Irvine, CA, United States). Frozen tissues were immediately submerged in lysis buffer and homogenized by steel bead beating at 1,000 rpm for 200 s using a Scientz-48L Frozen Tissue Grinder (Ningbo Xinzhi Biotechnology Co., Ltd., Zhejiang, China). Subsequent steps were performed following the manufacturer’s protocol. The DNA concentration and integrity were estimated by Quant-It dsDNA Assay (Life Technologies, Carlsbad, CA, United States) and a NanoDrop 2000 spectrophotometer (Thermo Fisher Scientific, Wilmington, DE, United States). Reactions (20 μL) contained 0.4 μL of FastPfu polymerase (Transgene, China), 10 ng of DNA, 0.8 mL of each primer (5 mM), 0.2 mL of BSA, 2 mL of dNTPs (2.5 mM), 4 mL of 5 × FastPfu buffer, and double-distilled water (ddH_2_O) to 20 mL. Primers 515F and 806R were used for bacterial V4 16S-rRNA amplification, while primers ITS1 and ITS2 were used for fungal ITS1 amplification. Reaction conditions for 16S amplification included an initial denaturation step at 95°C for 3 min, followed by 27 cycles at 95°C for 30 s, 55°C for 30 s, 72°C for 45 s, and a final extension at 72°C for 10 min. ITS1 amplification followed similar procedures but with 32 cycles. PCR products were assessed by 2% (w/v) agarose gel electrophoresis followed by staining with GelRed and visualization under ultraviolet light. The 16S rRNA and ITS2 regions were amplified in triplicate and mixed with DNA. Equal volumes were pooled for analysis using an Illumina MiSeq platform, and sequencing of the amplification library was completed by Sangon Biotech (Shanghai) Co., Ltd.

### Processing of Sequencing Data

To get high-quality clean reads, raw reads were further filtered according to the following rules using FASTP 0.20.0 (Chen et al., 2018): (i) removing reads containing more than 10% of unknown nucleotides (N); (ii) removing reads containing less than 50% of bases with quality (Q-value) >20. Paired end clean reads were merged as raw tags using FLASH v1.2.11 (Magoč and Salzberg, 2011) with a minimum overlap of 10 bp and mismatch error rates of 2%. High-quality effective sequences were analyzed with open-source Quantitative Insights into Microbial Ecology (QIIME 2.0) (Bolyen et al., 2019). The reads were denoised into amplicon sequence variants (ASVs) using the DADA2 pipeline which is tool available in QIIME 2.0 (Edgar, 2013). A representative sequence was selected from each OTU using default parameters. Bacterial reads were compared to the SILVA 138 database using a confidence threshold of 70%, while the UNITE v8.0 database was used for fungal reads (Nilsson et al., 2019). OTUs identified as unclassified bacteria or fungi at the phylum level, archaea, mitochondria, or chloroplasts were excluded. These were classified as additional quality controls or contaminants and removed before analysis. OTUs < 1% of average relative abundance in groups were categorized as “others.” OTUs < 0.001% of total sequences across all samples were discarded.

### Statistical Analysis

Lignocellulolytic enzyme activity was analyzed using a one-way analysis of variance (ANOVA). Differences between mean values were evaluated using the Tukey’s honestly significant difference (HSD) test. Alpha-diversity analysis was calculated for different gut groups using QIIME2.0 and visualized using the “vegan” (v2.5.3) R package (Caporaso et al., 2010). Shannon, Chao, Simpson, ACE, and coverage indices were calculated. Among these, Shannon and Chao indices were calculated for different groups, and the Wilcoxon rank test was used to calculate significant differences (*0.01 < *p* ≤ 0.05, ^**^0.001 < *p* ≤ 0.01). Beta diversity analysis was performed to investigate structural variation in microbial communities of the different group samples using Unweighted and Weighted UniFrac distance metrics (Lozupone and Knight, 2005) principal coordinates analysis (PCoA). The significance of differentiation of microbiota structure among groups were assessed by adonis or PERMANOVA (permutational multivariate analysis of variance) with 1,000 permutations (Jari Oksanen et al., 2019).

Bacterial and fungal taxa analyses were plotted using the “circos” R package (Krzywinski et al., 2009). Based on the modified OTU data, differences in the top ten genera relative abundances between different groups of microbial community species were compared by one-way ANOVA followed by the Scheffe test (*0.01 < *p* ≤ 0.05, ^**^0.001 < *p* ≤ 0.01), conducted using the “stats” R package.

Bacterial and fungal community functions were predicted by phylogenetic investigation of communities by reconstructing of unobserved states (PICRUSt2 v2.1.4) based on high-quality sequences (Langille et al., 2013). The PICRUSt package generates predictions from 16S rRNA and ITS2 data using annotations of sequenced genomes in the Greengene database and the Kyoto Encyclopedias of Genes and Genomes (KEGG, release 64.0) database.

## Results

### Gut Structure of *A. glabripennis* Larvae

**FIGURE 1:**
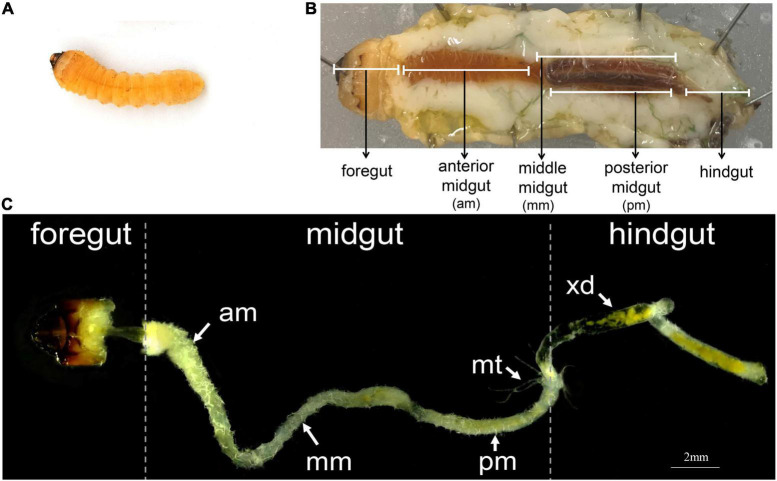
The digestive system of *A. glabripennis* larvae. **(A)** Second instar larvae, **(B)** Schematic diagram of the digestive system of *A. glabripennis* larvae during dissection. The division of the midgut refers to Mason et al. (2017). **(C)** Gut morphology of *A. glabripennis* larvae, separated into anterior midgut (am), middle midgut (mm), posterior midgut (pm), malpighian tubules (mt), xylem debris (xd).

The gut structure of *A. glabripennis* larvae accounted for the largest proportion of the body cavity except for the fat body (Figures 1A,B). It consists of three parts: the foregut, midgut, and hindgut, each of which can be separated (Figures 1B,C). The foregut is short and only extends to the end of the prothorax. The midgut is further subdivided into three parts: the anterior midgut (am), middle midgut (mm), and posterior midgut (pm). In general, the anterior end of the midgut is enlarged, starts from the anterior end of the mid thorax, extends to the eighth abdominal segment, and then folds forward, turning at the second abdominal segment and extending to the eighth abdominal segment. The hindgut is relatively thin, starting from the eighth abdominal segment, reaching the middle of the ninth abdominal segment, extending forward to the seventh abdominal segment, and finally folding toward the anus. Malpighian tubules branch from the midgut-hindgut border (Figure 1C). We also observed digested xylem debris in the gut (Figure 1C).

### Lignocellulose-Degrading Enzyme Activities in the Gut of *A. glabripennis* Larvae

**FIGURE 2:**
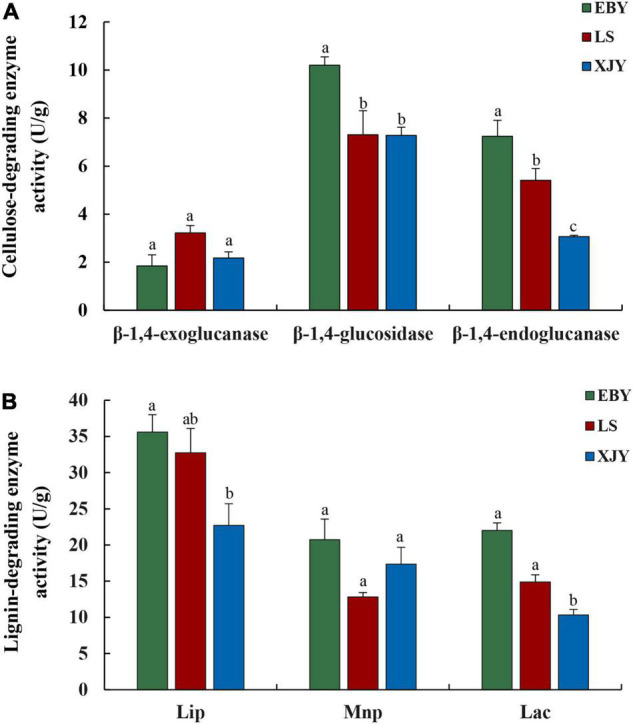
Gut enzyme activity of *A. glabripennis* larvae fed on different *Populus gansuensis* (EBY), *Salix babylonica* (LS), or *Populus alba* var. *pyramidalis* (XJY) as host tree species. **(A)** Cellulose-degrading enzyme activity in the larval gut. **(B)** Lignin-degrading enzyme activity in the larval gut. LiP, lignin peroxidase; MnP, manganese peroxidase; Lac, laccase. Results followed by different letters are significantly different according to the HSD test (*p* ≤ 0.05).

The enzyme activities of three different types of cellulases were compared between larvae reared on different tree species (Figure 2A). The β-1,4-glucosidase and β-1,4-endoglucanase activities were significantly higher in enzyme extracts from the guts of *A. glabripennis* fed on EBY than those fed on LS and XJY (*p* < 0.001). In addition, β-1,4-endoglucanase activity was also significantly higher in LS gut extracts than in XJY extracts (*p* < 0.01). The β-1,4-exoglucanase activity was highest in LS but was not significantly different from the other two species (*p* > 0.05; Figure 2A). The enzyme degradation of lignin by enzyme extracts from the guts of *A. glabripennis* showed that LiP and Lac activities were higher in EBY than in XJY and LS, but there was no significant difference with LS (Figure 2B). Lac activity was also significantly higher in LS gut extracts than in XJY extracts (*p* < 0.01). There were no significant differences in MnP activities in gut extracts from larvae fed on the three different host trees (*p* > 0.05; Figure 2B).

### Bacterial and Fungal Communities of *A. glabripennis* Larval Guts Fed on Different Host Tree Species

A total of 1,006,740 paired-end reads of 16S rRNA V3-V4 amplicon sequences and 1,027,726 reads of ITS amplicon sequences were generated to survey the bacterial and fungal communities, respectively. After quality filtering, we obtained 984,910/992,800 high-quality sequences (an average of 108,222/110,311 reads per sample), from which 2,856/494 OTUs were identified from 9 samples (Supplementary Table 1). The OTU-level rarefaction curves were generated to compare the richness and evenness of OTUs among samples (Supplementary Figures 1, 2), indicating that these specimens’ sequencing depths were appropriate. Bacterial and fungal OTU richness and diversity varied in the guts of *A. glabripennis* larvae fed on different host tree species. Larvae obtained from EBY had a greater richness of gut bacterial and fungi OTUs than larvae from LS and XJY (Supplementary Table 2). LS larvae guts had the fewest bacterial OTUs, while XJY larvae guts had the fewest fungal OTUs. Venn diagrams were plotted to visualize the shared and unique OTUs among the three different host trees. A total of 15.38% of bacterial OTUs were shared among the three groups, with unique OTUs mainly being present in EBY (23.46%), LS (20.5%), and XJY (21.02%; Supplementary Figure 3). A total of 19.13% of fungal OTUs were shared among the three groups, with unique OTUs largely being present in EBY (21.6%), LS (16.07%), and XJY (10.71%; Supplementary Figure 4).

**FIGURE 3:**
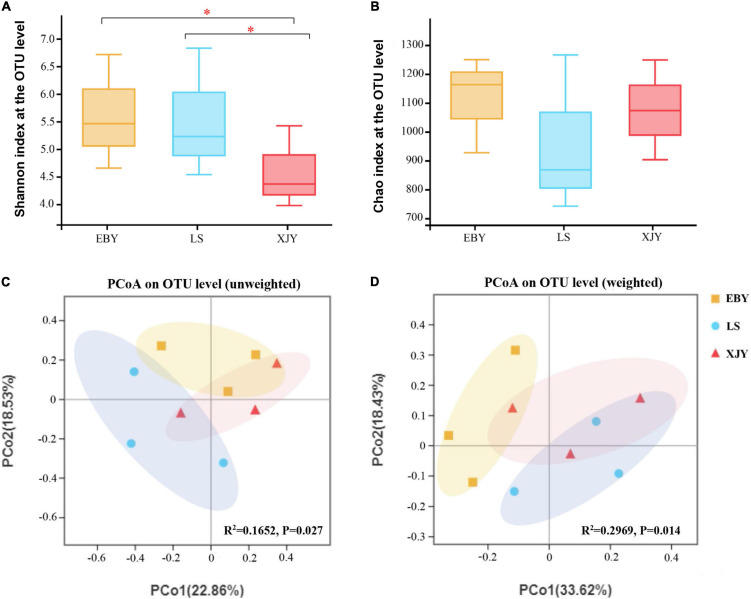
Bacterial communities in the *A. glabripenni*s larval gut. Boxplots of **(A)** species diversity (Shannon index) and **(B)** species richness (Chao index). Significant differences in alpha-diversity were analyzed by Wilcoxon rank-sum test (*0.01 < *p* ≤ 0.05). **(C)** Unweighted and **(D)** weighted UniFrac-based PCoA plots of bacterial communities. The significant differences in beta-diversities were analyzed using adonis analysis with 1,000 Monte Carlo permutations. EBY, *Populus gansuensis*; LS, *Salix babylonica*; XJY, *Populus alba* var. *pyramidalis*.

For the observed OTUs, alpha-diversity was used to evaluate differences in community richness (Chao index) and diversity (Shannon index) among the guts of larvae fed on different tree species. The results showed no significant differences in Chao index for gut bacterial microbiota between different host trees (Figure 3B). However, there were significant differences in gut microbiota diversity for the three tree species (*p* < 0.05). Specifically, the Shannon index of gut bacterial microbiota in EBY and LS was significantly higher than that of XJY (Figure 3A). A significant effect of different host tree was observed for bacterial community membership when using PCoA based on unweighted and weighted UniFrac distances (Figures 3C,D, *R*^2^ = 0.1652, and *p* = 0.027 for unweighted; *R*^2^ = 0.2969 and *p* = 0.014 for weighted). The bacterial composition of LS was more similar to XJY than EBY.

**FIGURE 4:**
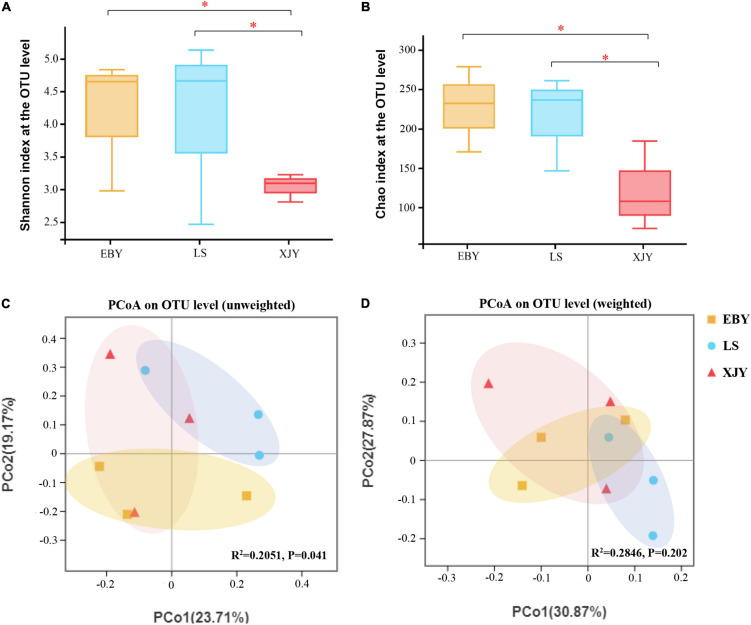
Fungal communities in the *A. glabripennis* larval gut. Boxplots of **(A)** species diversity (Shannon index) and **(B)** species richness (Chao index). Significant differences in alpha-diversity were analyzed by Wilcoxon rank-sum test (*0.01 < *p* ≤ 0.05). **(C)** Unweighted and **(D)** weighted UniFrac-based PCoA plots of fungal communities. The significant differences in beta-diversities were analyzed using adonis analysis with 1,000 Monte Carlo permutations. EBY, *Populus gansuensis*; LS, *Salix babylonica*; XJY, *Populus alba* var. *pyramidalis*.

The fungal composition in larvae guts was simpler than bacteria; both the number of observed OTUs and the Shannon index was lower for fungi than bacteria (Supplementary Table 2 and Figure 4). The trends in the Chao index and the Shannon index were similar. Alpha-diversity was significantly higher in EBY and LS samples compared with XJY samples (*p* < 0.05; Figures 4A,B). However, we also found that fungal species richness and species diversity were not significantly different between EBY and LS. A significant effect of different host trees was observed for fungal community membership when using unweighted UniFrac distances (Figure 4C, *R*^2^ = 0.2051, and *p* = 0.041). No significant differences were observed when considering weighted UniFrac distances (Figure 4D, *R^2^* = 0.2846, and *p* = 0.202). Comparatively, the fungal communities of EBY and XJY were more similar than those of LS.

**FIGURE 5:**
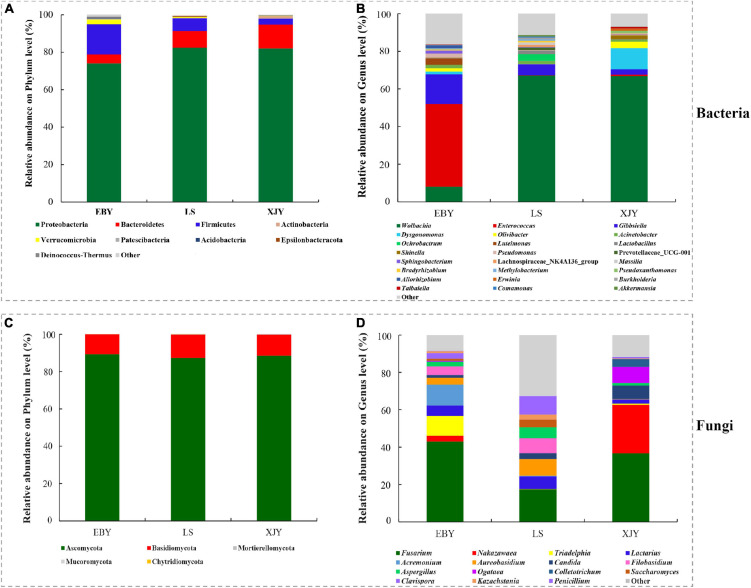
Taxonomic composition of bacterial and fungal communities associated with the *A. glabripennis* larval gut. The relative abundance is shown for each bacterial **(A)** phylum and **(B)** genus. The relative abundance is also shown for each fungal **(C)** phylum and **(D)** genus. Each bar is indicated by a different color at the phylum and genus level. OTUs < 1% of the average relative abundance in groups are summarized as “others”. EBY, *Populus gansuensis*; LS, *Salix babylonica*; XJY, *Populus alba* var. *pyramidalis*.

**FIGURE 6:**
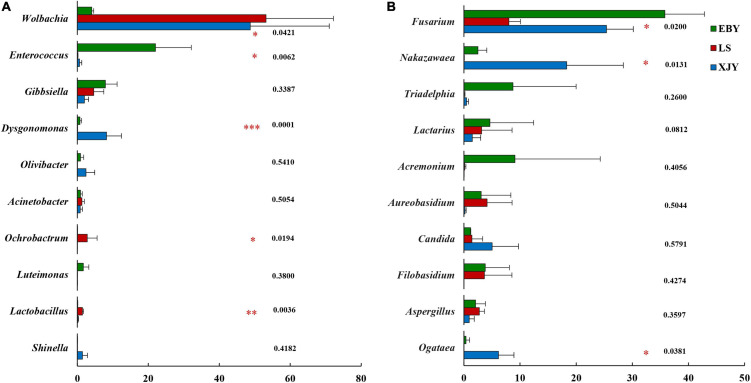
Significant differences in microbial composition in the larval gut following feeding on three host tree species. Differences in relative abundance are shown for the ten most significant differences. Relative abundances are shown for **(A)** bacteria and **(B)** fungi. EBY, *Populus gansuensis*; LS, *Salix babylonica*; XJY, *Populus alba* var. *pyramidalis*. One-way ANOVA followed by Scheffe test; *0.01 < *p* ≤ 0.05, ^**^0.001 < *p* ≤ 0.01, ^***^p ≤ 0.001.

The relative abundance of bacterial and fungal communities in larval guts from the three host trees was examined at phylum and genus levels (Figure 5). Across all samples, the dominant component of the bacterial community at the phylum level was Proteobacteria, followed by Bacteroidetes, Firmicutes, Actinobacteria, Verrucomicrobia, Patescibacteria, Acidobacteria, Epsilonbacteraeota, and Deinococcus-Thermus (Figure 5A). *Wolbachia* was dominant in larval gut communities at the genus level, followed by *Enterococcus*, *Gibbsiella*, *Dysgonomonas*, *Olivibacter*, *Acinetobacter*, *Ochrobactrum*, *Luteimonas*, *Lactobacillus*, *Shinella*, and *Pseudomonas* (Figure 5B). However, each group had a significantly enriched set of microorganisms at the genus level (Figure 6A and Supplementary Figure 5). *Wolbachia* was notably enriched in the guts of larvae fed on LS and XJY compared with EBY (*p* < 0.05), whereas *Enterococcus* and *Gibbsiella* were the most abundant bacteria in EBY (*p* < 0.01). *Dysgonomonas* was notably enriched in XJY (*p* < 0.001), while *Ochrobactrum* and *Lactobacillus* were significantly enriched in LS (*p* < 0.05).

Ascomycota and Basidiomycota mainly dominated the fungal communities in larvae of *A. glabripennis* at the phylum level (Figure 5C). Across all samples, the highest proportion of the fungal community at the genus level was *Fusarium* (species-level analysis identified *Fusarium solani*), followed by *Nakazawaea, Triadelphia*, *Lactarius*, *Acremonium*, *Aureobasidium*, *Candida*, *Filobasidium*, *Aspergillus*, *Ogataea*, *Colletotrichum*, *Saccharomyces*, *Clavispora*, *Kazachstania*, and *Penicillium* (Figure 5D and Supplementary Figure 6). There were more unclassified fungi at the genus level in LS than in EBY and XJY samples (Supplementary Figure 6). The top ten fungal genera inhabiting the larval guts also varied between host tree species (Figure 6B). For instance, *Fusarium* was notably enriched in EBY and XJY compared with LS (*p* < 0.05), whereas *Nakazawaea* and *Ogataea* were the most abundant fungi in XJY (*p* < 0.05). The presence of common genera suggests that they might perform essential functions in the growth and development of *A. glabripennis* larvae, especially *Fusarium* among fungi and *Wolbachia* among bacteria (Supplementary Figures 5, 6). In addition, most of the highly abundant bacterial and fungal genera were correlated to varying degrees; the relative abundance of *Fusarium* was negatively correlated with *Wolbachia* (Supplementary Figure 7).

### Microbial Functions in *A. glabripennis* Larval Guts Predicted by PICRUSt2

**FIGURE 7:**
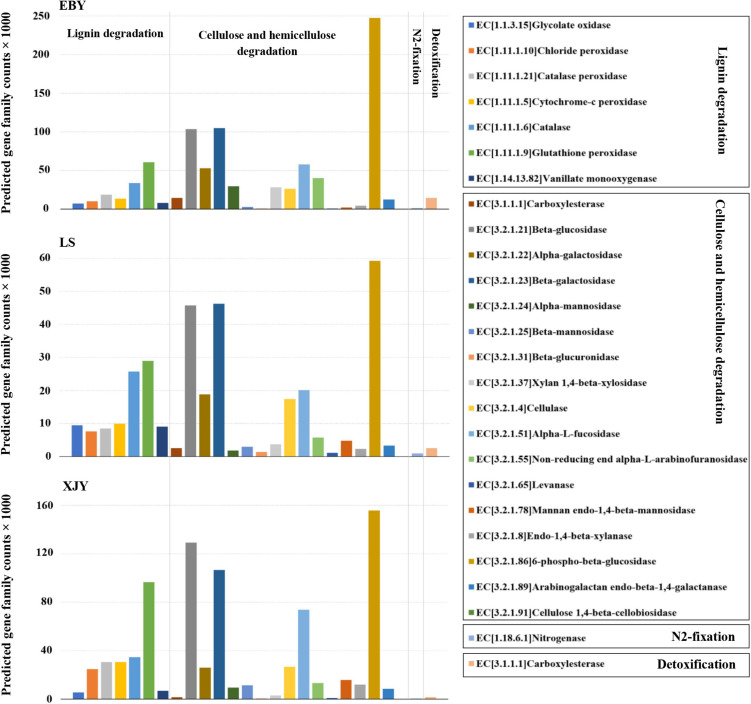
Predicted bacterial community functions in the *A. glabripennis* larval gut following feeding on different host tree species. The total number of predicted gene family counts is shown. EBY, *Populus gansuensis*; LS, *Salix babylonica*; XJY, *Populus alba* var. *pyramidalis*.

Bacterial community functional prediction was performed on the guts of larvae fed on the three host trees, and 24 genes potentially related to lignocellulose degradation were identified, along with one gene putatively associated with nitrogen fixation and one gene possibly involved in detoxification (Figure 7). The detailed enzyme-catalyzed reactions of these gene products are shown in Supplementary Table 3. Based on lignocellulose degradation pathways, 17 predicted genes were involved in cellulose and hemicellulose degradation, of which 6-phospho-β-glucosidase (EC3.2.1.86) was the most abundant in the guts of larvae fed on all three different host tree sources, followed by β-glucosidase (EC3.2.1.21), β-galactosidase (EC3.2.1.23), and alpha-L-fucosidase (EC3.2.1.51; Figure 7). 6-phospho-β-glucosidase (EC3.2.1.86) was significantly higher in EBY compared with those in LS and XJY (*p* < 0.05). β-glucosidase (EC3.2.1.21) and β-galactosidase (EC3.2.1.23) were significantly higher in EBY and XJY than in LS (*p* < 0.05; Supplementary Table 4). Seven predicted genes were involved in lignin degradation, the most significant of which were glutathione peroxidase (EC1.11.1.9) and catalase (EC1.11.1.6), which were significantly higher in XJY compared to the other two tree species (*p* < 0.05; Supplementary Table 4). For biological nitrogen fixation, we predicted key genes for the nitrogenase component protein (nifH). We predicted a key gene encoding a carboxylesterase component protein (CarE) for insect detoxification. Nitrogenase (EC1.18.6.1) and carboxylesterase (EC3.1.1.1) were highest in EBY but were not significantly different from the other two species (*p* > 0.05; Supplementary Table 4).

**FIGURE 8:**
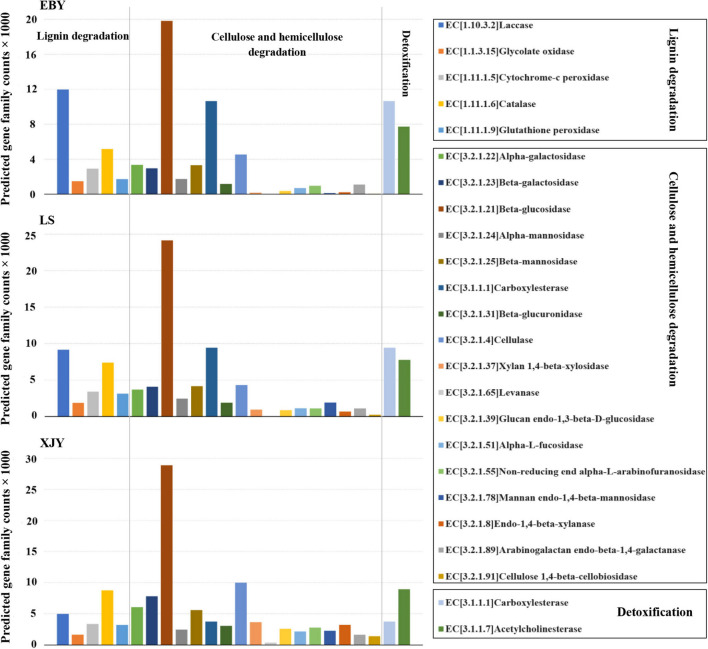
Predicted fungal community functions in the *A. glabripennis* larval gut following feeding on different host tree species. The total number of predicted gene family counts is shown. EBY, *Populus gansuensis*; LS, *Salix babylonica*; XJY, *Populus alba* var. *pyramidalis*.

Fungal community functional prediction identified 22 genes potentially related to lignocellulose degradation and two genes potentially related to detoxification (Figure 8). The detailed enzyme-catalyzed reactions are shown in Supplementary Table 5. Most of the predicted genes were involved in cellulose and hemicellulose degradation, among which β-glucosidase (EC3.2.1.21) was the most abundant, but there was no significant difference among the three host tree species (*p* > 0.05; Supplementary Table 6). Five predicted genes were involved in lignin degradation, of which laccase (EC1.10.3.2) was the most abundant. In addition, it was also significantly higher in EBY than in XJY and LS (*p* < 0.05; Supplementary Table 6). The predicted genes involved in detoxification degradation carboxylesterase (EC3.1.1.1) and acetylcholinesterase (EC3.1.1.7).

## Discussion

Herbivorous insects feed on different host plants, leading to differences in their gut structures. The digestive tract of *A. glabripennis* larvae is divided into three parts, the foregut, midgut, and hindgut, each of which can be separated. The midgut forms a single loop under itself (Figure 1). The gut structure of *A. glabripennis* is similar to that of the wood borer pest *Apriliona germari* (Wang, 2012). The digestive tract of the beetle larvae is larger than the body length, which is significantly different from other insects (Wang, 2012; Wu et al., 2016), such as wasps (Foelker, 2016). In addition, the midgut of *A. glabripennis* larvae comprises > 90% of the total length of the digestive tract and is further divided into the anterior midgut, middle midgut, and posterior midgut (Mason et al., 2017). The midgut of *Eucryptorrhynchus scrobiculatus* and *Eucryptorrhynchus brandti* accounted for 39.63% and 36.91% of the entire digestive tract, respectively. According to the shape of the midgut, it was divided into two parts: the anterior midgut and the posterior midgut (Chen, 2016).

Previous research suggested that lignin, cellulose, and hemicellulose degradation occur within the gut of *A. glabripennis* larvae (Geib et al., 2008, 2009, 2010). Studies on midgut transcriptome data from *A. glabripennis* larvae revealed a number of enzymes with putative roles in the digestion of xylem, detoxification, and nutrient extraction, which likely contribute to the ability of these larvae to thrive in a broad range of host trees (Scully et al., 2013a,^2018^). In the present study, feeding on the preferred host tree species (*P. gansuensis*) resulted in high lignocellulose enzyme activity, whereas feeding on *P. alba* var. *pyramidalis*, characterized by strong resistance, lignocellulose enzyme activity was lowest (Figure 2). This result is consistent with previous studies by Geib et al. (2010) and Li et al. (2010). In addition, Chen et al. (2006) concluded that the order of cellulase activities in the guts of *A. glabripennis* larvae was β-1,4-glucosidase > β-1,4-endoglucanase > β-1,4-exoglucanase, consistent with our current results. However, the activities of lignocellulose enzymes in the guts of different species of longhorn beetle larvae are not the same. Yin et al. (1996) found that the β-1,4-endoglucanase activity of larvae of *Apriona germari* was highest, while Suo et al. (2004) found that the activity of β-1,4-exoglucanase was highest in the larvae of *Monochamus alternatus*. Therefore, the activities of insect digestive enzymes may be affected by many factors, including the host environment, insect taxa, developmental state of the insect body, mobility, energy requirements, and even genes (Wang et al., 2020).

Wood-feeding insect guts have a limited capacity to digest lignocellulose, and they often work in collaboration with the gut microbiota to degrade lignin biopolymers and release glucose (Brune and Dietrich, 2015). The *A. glabripennis* larval gut harbors a rich diversity of microbes, with a marked variation in community complexity and composition when feeding on different host trees (Geib et al., 2010). When feeding on the preferred host tree, *A. glabripennis* larval guts had the most diverse bacterial and fungal community, followed by the alternative host tree species, and the least diverse microbial community was associated with the high resistance host tree (Supplementary Figures 3, 4). This is consistent with the results of lignocellulose activity in larval gut extracts fed on the three host tree species. The results show that the host tree can impact gut microbial community complexity and lignocellulose activity in *A. glabripennis* (Geib et al., 2010; Scully et al., 2018).

The bacterial community complexity and composition of the *A. glabripennis* larval gut were influenced by host tree species and geographic location. Larvae fed on EBY had the greatest diversity of any 16S library, with the highest number of OTUs and the highest Chao richness estimate (Scully et al., 2018; Mason et al., 2019). At the phylum level, characterization of *A. glabripennis* larval gut bacterial communities of insects collected from an invasion area in New York, United States, and from the native range in Hebei, China, gave similar community complexity results to those of our current study, with Proteobacteria, Bacteroidetes, Firmicutes, and Actinobacteria as the dominant bacteria (Schloss et al., 2006; Geib et al., 2010; Podgwaite et al., 2013), but the bacterial diversity in the gut of larvae from different regions was significantly different at the genus taxonomic level. In the present study, *Wolbachia* dominated the guts of larvae feeding on LS and XJY, while *Enterococcus* and *Gibbsiella* were the most abundant in the guts of larvae feeding on EBY. In the United States, identification of bacterial taxa (OTUs) from the larval gut of *A. glabripennis* fed on three different host trees showed that *Enterobacter* was the dominant bacterial genus in Pin oak and Callery pear, while *Enterococcus* was dominant in sugar maple (Geib et al., 2010). Furthermore, sugar maple and EBY were the preferred host species of *A. glabripennis*, and *Enterococcus* was the most dominant genus in the gut of larvae feeding on these trees. However, the relationship between *Enterococcus* and feeding on different host trees cannot be determined for larvae of *A. glabripennis*; this will be studied in future work to explore host tree resistance to cerambycids.

In addition, *Wolbachia* is the widest endosymbionts within the insect cells, with an infection rate of ∼66% in all species of insects, and it has a great impact on the growth and development of host insects (Ahmed et al., 2016). Although *Wolbachia* was found in the gut of *Saperda populnea* larvae in previous work (Cao et al., 2021), the present study was the first to show that *Wolbachia* is abundant in the gut of *A. glabripennis*. Notably, total DNA was extracted using larval gut tissue (containing the contents) in this study. The relative abundance of *Wolbachia* in larval gut microbiotas fed on resistant and alternative hosts was significantly higher than that in preferred hosts (Figures 5, 6). This may suggest that the gut bacterial communities may have low numbers and have less possibility to exert effects in resistant and alternative hosts, which requires more research to elaborate on this finding.

In addition to ubiquitous associations with bacteria, wood-feeding insects often have associations with eukaryotic partners. Our current study revealed significant differences in fungal diversity in the guts of larvae feeding on different host tree species, and gut fungal diversity decreased with increasing host resistance capacity (Supplementary Figure 4). In previous research, the frass component of ovipositing behavior, the presence of *F. solani* (FSSC) in the oviposition pits but not nearby tissues, and the consistent detection of *F. solani* across studies in space and time suggested that this fungus plays a vital role in the life history of *A. glabripennis* (Scully et al., 2013a,b; Mason et al., 2019). This is consistent with our current findings; fungal communities in the *A. glabripennis* larval gut were dominated by *F. solani* following feeding on all three host trees. We did not observe FSSC in any adjacent healthy phloem tissue, but we did detect FSSC in 100% of oviposition pits in China (unpublished data). Besides *F. solani*, the guts of larvae feeding on different host trees contained unique genera of dominant fungi; the major fungal genera in larval guts fed on XJY also included *Nakazawaea*, *Ogataea*, and *Candida*, while *Triadelphia* and *Acremonium* were mainly found in EBY, and *Penicillium* was detected in LS. The reason for this discrepancy is not clear, but it could reflect differences in larval health and/or host tree species.

The xylem is mainly made of lignin, cellulose, and hemicellulose. Different digestive enzymes are required for different substrates, which may sometimes act incongruence (Chiappini and Aldini, 2011). For a long time, there have been different opinions about the source of digestive enzymes in wood-boring pests. These lignocellulolytic enzymes may originate from gut symbionts, ingestion of enzymes produced by wood decay fungi, the insect itself, or some combination of these (Martin, 1983; Brune, 2003; Suh et al., 2005). In the present study, we predicted several genes encoding enzymes in the gut microbiota involved in lignin degradation, including several peroxidases, laccases, enzymes oxidizing phenolic/non-phenolic compounds, and lignin-modifying proteins (Levasseur et al., 2013). These include gluco-oligosaccharide oxidases, which oxidize different carbohydrates, and glycolate oxidase (van Hellemond et al., 2006), which oxidizes glycolate to glyoxylate and generates reactive oxygen species. These gene family counts were higher in the gut of larvae feeding on different host tree species (Figures 7, 8). This suggests that larval gut microbial communities feeding on different host trees have endogenous potential to degrade lignin and extract nutrients from woody tissue (Scully et al., 2012). Previous work suggested that a gut-derived *F. solani* isolate could detect laccases that degrade lignin depolymerization, but lignin- and Mn-dependent peroxidase activities were not detected. In this study. *F. solani* was the most dominant fungus in the gut of larvae feeding on different hosts. Interestingly, our current study echoes previous findings: laccase activity was among the most pronounced gut fungal community functions.

Regarding degradation of cellulose and hemicellulose, there are genes encoding enzymes, such as β-glucosidase which hydrolyzes cellobiose and short-chain oligosaccharides (Glass et al., 2013), cellulase, which cleaves internal bonds in cellulose (Klyosov, 1990), and β-galactosidase, which hydrolyzes β-galactosidic bonds (Husain, 2010). In the present study, we identified genes that encode enzymes involved in the degradation of cellulose and hemicellulose in the gut microbial community, including β-galactosidase (Husain, 2010), β-glucosidase (Glass et al., 2013), xylan1,4-β-xylosidase (Zhou et al., 2012), alpha-L-fucosidase, endo1,4-b-xylanase, β-mannosidase, carboxylesterase, and others (Numan and Bhosle, 2006). These gene family counts were higher in the gut of larvae feeding on different host tree species (Figures 7, 8). Microbial community composition varies between populations and host trees but appears to perform similar functional roles (Scully et al., 2013a,b, 2018). Moreover, predicted genes were involved in cellulose and hemicellulose degradation was the highest in preferred host trees (Supplementary Tables 4, 6), which may be related to the higher microbial diversity in larval gut feeding on preferred host trees than in the other two tree species. Most of the genes predicted in the present work were detected previously in the midgut transcriptomes of *A. glabripennis* and *Trichoferus campestris* (Scully et al., 2013a; Mohammed et al., 2018), including nifH involved in nitrogen fixation and providing nitrogen to *A. glabripennis* larval gut bacterial communities, as reported by Thompson et al. (2013, 2014) and Gaby and Buckley (2014). We also identified carboxylesterases and acetylcholinesterase which might be involved in detoxification metabolism, which play an important role in catabolizing plant secondary metabolites and other exogenous toxins and maintaining the normal physiological and biochemical activities of insects.

## Conclusion

In summary, this study described the *A. glabripennis* larval gut structure, examined gut lignocellulose activities in tree hosts with different resistance levels, and predicted the roles of the gut microbiota in the survival of larvae in nutrient-deficient host xylem. The digestive tract of *A. glabripennis* larvae is divided into the foregut, midgut, and hindgut. The midgut forms a single loop under itself and comprises > 90% of the total length of the digestive tract. The microbial community composition and lignocellulose activity of gut extracts from larvae correlated well with host tree species. When feeding on preferred host trees, *P. gansuensis* exhibited high microbial community diversity and lignocellulose enzyme activity compared with feeding on an alternative host (*S. babylonica*) and a highly resistant host (*P. alba* var. *pyramidalis*). *Wolbachia* was most abundant in the gut of *A. glabripennis*, but less abundant in the gut of larvae feeding on the preferred host than on other trees. The *A. glabripennis* larval gut is consistently associated with *F. solani*. The functional predictions of microbial communities in the larval gut fed on different resistant host trees suggested that they all have roles in degrading lignocellulose, detoxification, and fixing nitrogen. These functions likely contribute to the ability of these larvae to thrive in a broad range of host tree species.

## Data Availability Statement

The datasets presented in this study can be found in online repositories. The names of the repository/repositories and accession number(s) can be found below: PRJNA815744.

## Author Contributions

LW and YL contributed to the design of the study. CL, XW, and GW collected the samples. LW and SS observed the larval gut structure. LW analyzed the data. LW, ZD, and CL wrote and reviewed the manuscript. All authors have read and agreed to the published version of the manuscript.

## Conflict of Interest

The authors declare that the research was conducted in the absence of any commercial or financial relationships that could be construed as a potential conflict of interest.

## Publisher’s Note

All claims expressed in this article are solely those of the authors and do not necessarily represent those of their affiliated organizations, or those of the publisher, the editors and the reviewers. Any product that may be evaluated in this article, or claim that may be made by its manufacturer, is not guaranteed or endorsed by the publisher.
