# Supplementary material for: Gut Lignocellulose Activity and Microbiota in Asian Longhorned Beetle and Their Predicted Contribution to Larval Nutrition
Source: Front Microbiol. 2022 May 9;13:899865. doi: 10.3389/fmicb.2022.899865 (PMC9124977; doi:10.3389/fmicb.2022.899865)
Supplement: Supplementary file 1 [file Data_Sheet_1.docx]

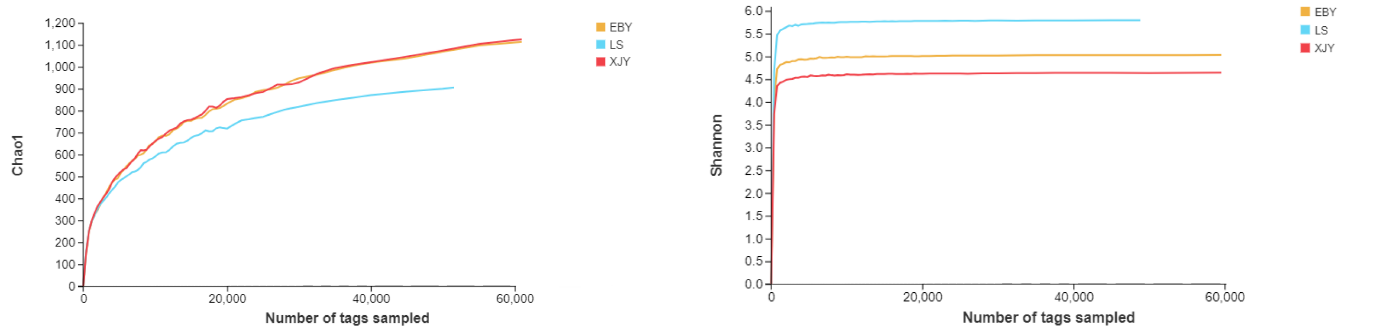


**Figure S1. Rarefaction curves of gut bacterial communities.** Rarefaction analysis involved random sampling with replacement and estimation of the total number of OTUs present in the samples. The curves reach asymptote when observed diversity or richness is saturated based on the number of sequences that are sub-sampled. (A) Chao index and (B) Shannon index. EBY, *Populus gansuensis*; LS, *Salix babylonica*; XJY, *Populus alba* var. *pyramidalis*.


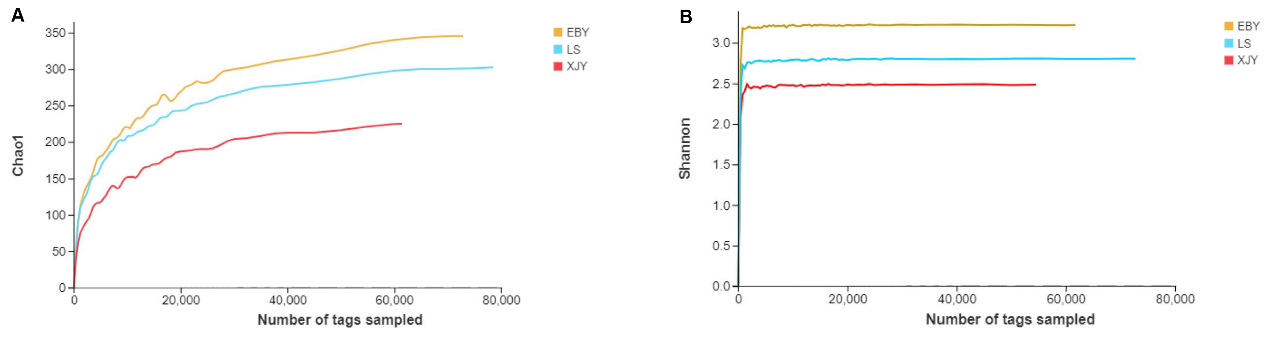


**Figure S2. Rarefaction curves of gut fungal communities.** Rarefaction analysis involved random sampling with replacement and estimation of the total number of OTUs present in the samples. The curves reach asymptote when observed diversity or richness is saturated based on the number of sequences that are sub-sampled. (A) Chao index and (B) Shannon index. EBY, *Populus gansuensis*; LS, *Salix babylonica*; XJY, *Populus alba* var. *pyramidalis*.


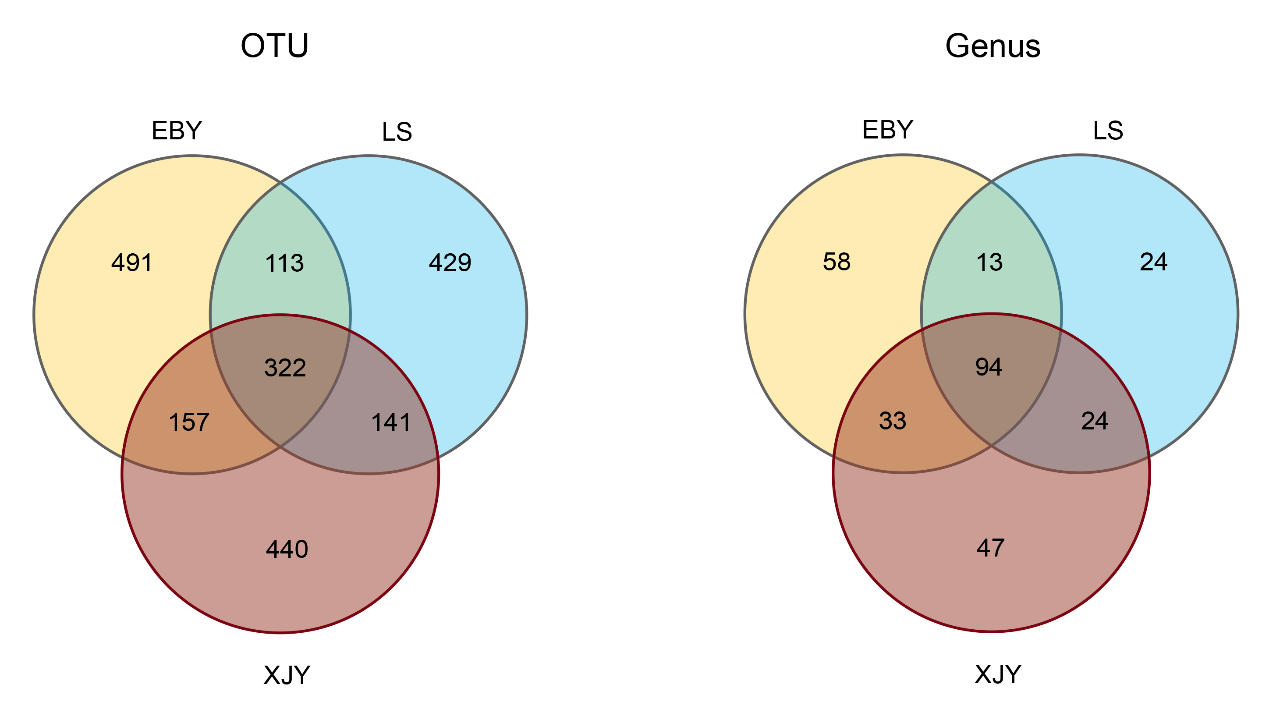


**Figure S3. Venn diagram of shared and unique OTUs in the gut bacterial communities of the *A. glabripennis* larvae from different host trees.** EBY, *Populus gansuensis*; LS, *Salix babylonica*; XJY, *Populus alba* var. *pyramidalis*.


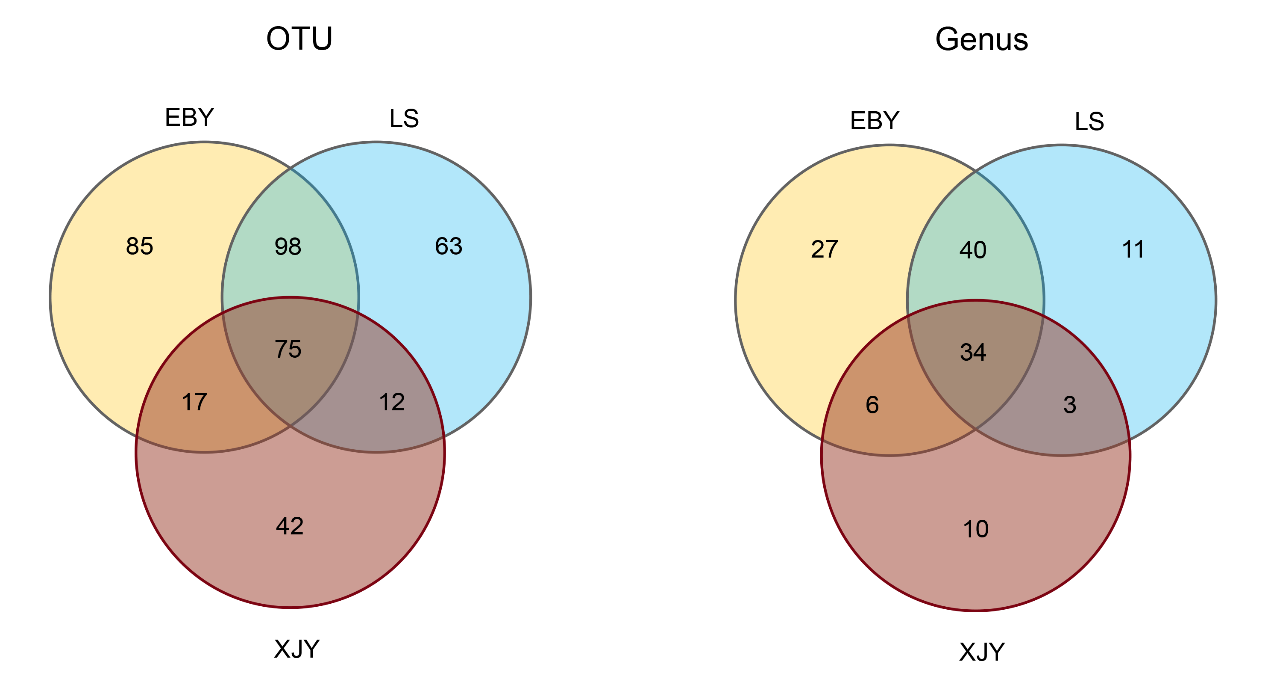


**Figure S4. Venn diagram of shared and unique OTUs in the gut fungal communities of the *A. glabripennis* larvae from different host trees.** EBY, *Populus gansuensis*; LS, *Salix babylonica*; XJY, *Populus alba* var. *pyramidalis*.


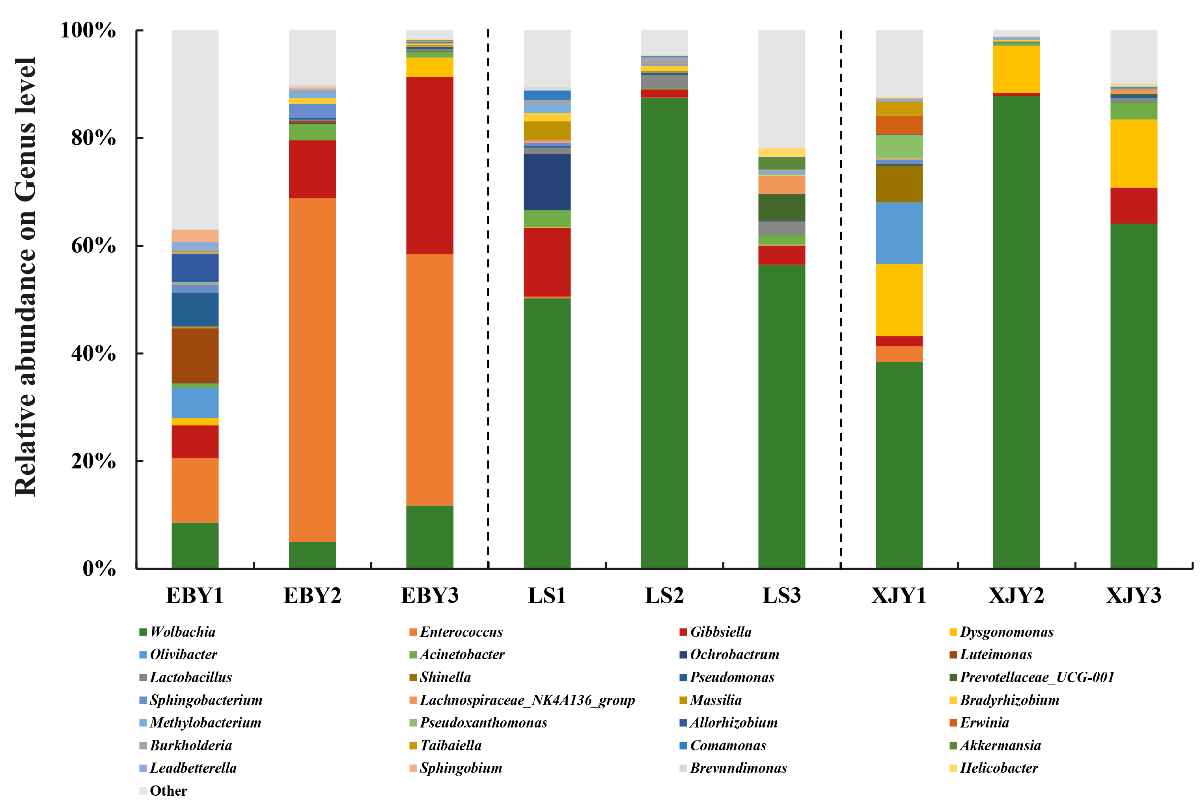


**Figure S5. Relative abundance of bacterial communities associated with the *A. glabripennis* larval gut from different host trees.** EBY, *Populus gansuensis*; LS, *Salix babylonica*; XJY, *Populus alba* var. *pyramidalis*.


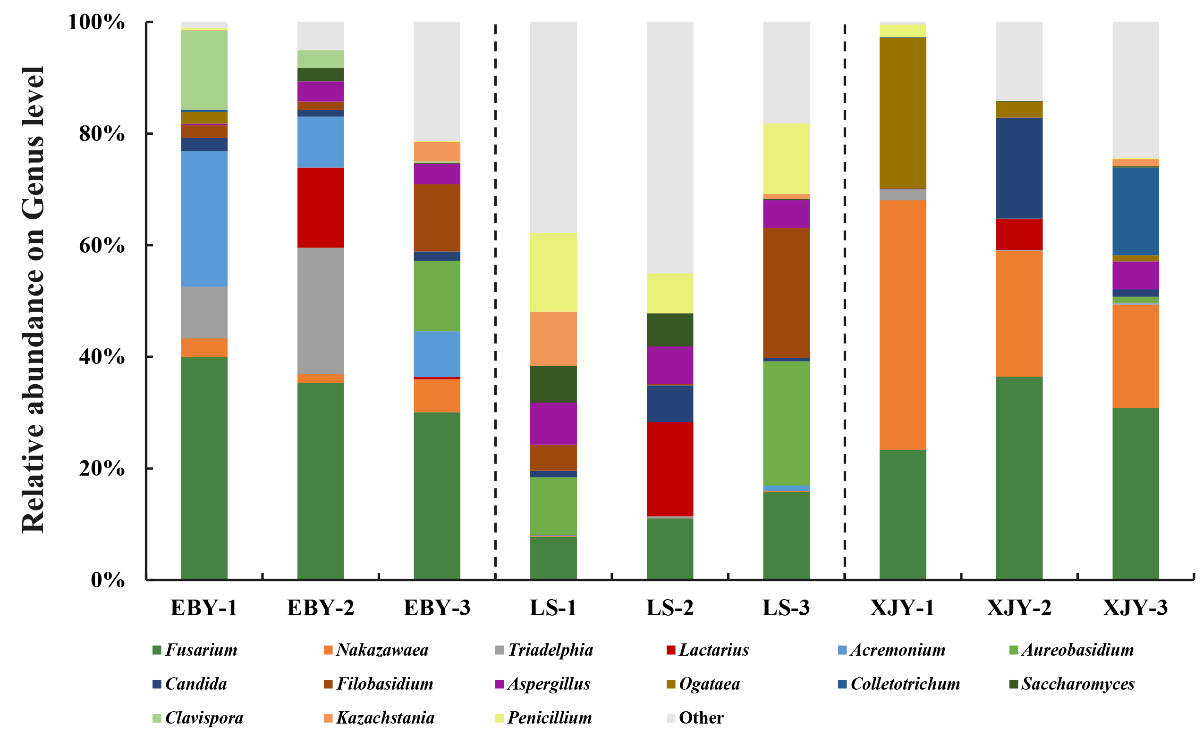


**Figure S6. Relative abundance of fungal communities associated with the *A. glabripennis* larval gut from different host trees.** EBY, *Populus gansuensis*; LS, *Salix babylonica*; XJY, *Populus alba* var. *pyramidalis*.


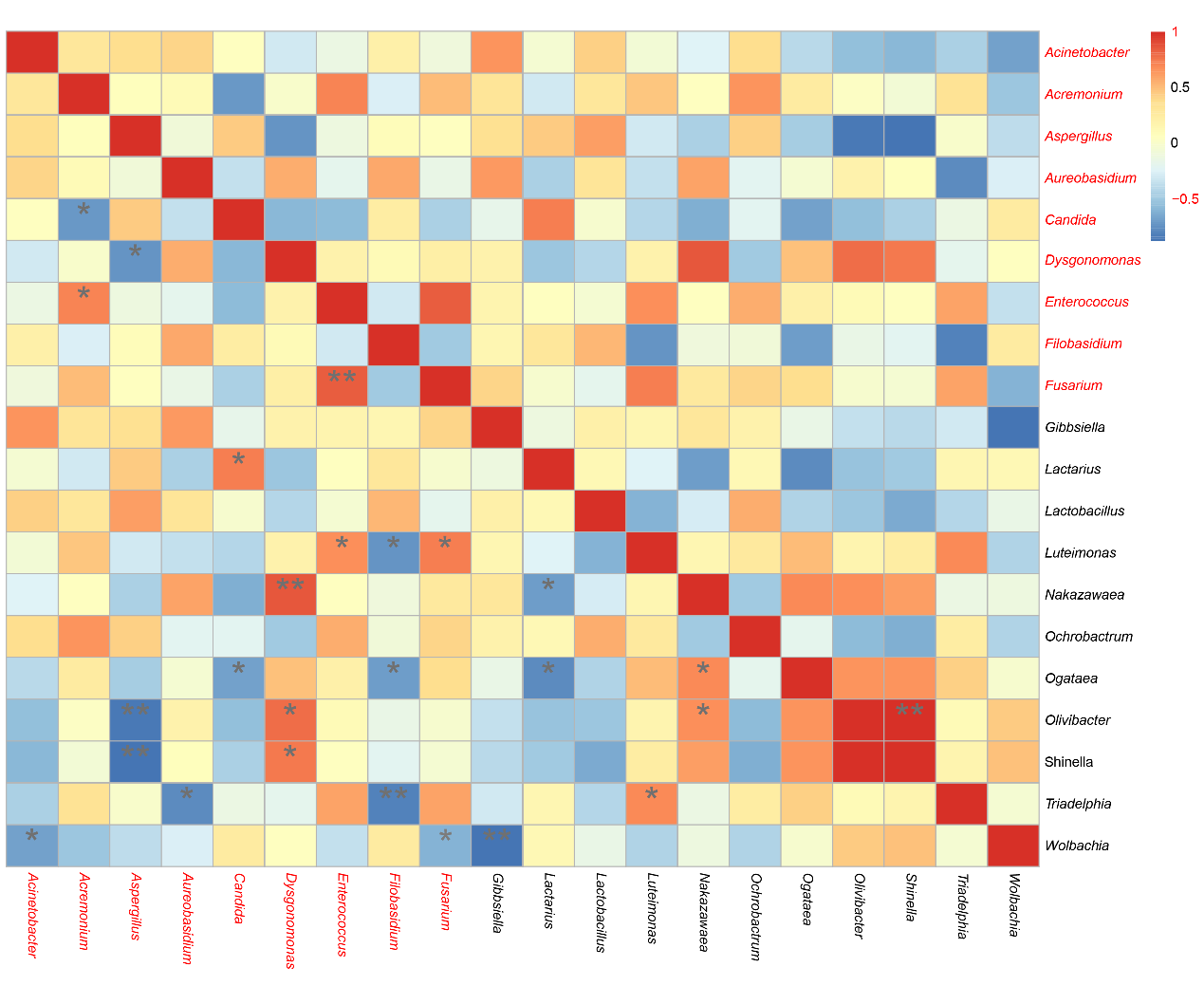


**Figure S7. Spearman correlations of samples with the OTU abundance of bacterial and top ten fungal genera in *A. glabripennis* larval gut.**

**Table S1. Summary statistics for the Illumina MiSeq runs for all samples**

| Sample ID | **16S RNA gene** | | |  | **ITS region** | | |
| --- | --- | --- | --- | --- | --- | --- | --- |
|  | Mean length | Number of received reads | Number of filtered reads |  | Mean length | Number of received reads | Number of filtered reads |
| EBY1 | 443 | 118419 | 115259 (97%) |  | 386 | 127011 | 122970 (97%) |
| EBY2 | 466 | 111635 | 110206 (99%) |  | 368 | 111943 | 107950 (96%) |
| EBY3 | 466 | 112285 | 111290 (99%) |  | 375 | 108073 | 104946 (97%) |
| LS1 | 466 | 108520 | 107002 (99%) |  | 339 | 103690 | 100109 (97%) |
| LS2 | 441 | 106676 | 102847 (96%) |  | 381 | 118575 | 115398 (97%) |
| LS3 | 441 | 106130 | 102820 (97%) |  | 390 | 122843 | 119160 (97%) |
| XJY1 | 461 | 108184 | 106364 (98%) |  | 375 | 108352 | 104144 (96%) |
| XJY2 | 441 | 114032 | 109995 (96%) |  | 331 | 112349 | 108380 (96%) |
| XJY3 | 466 | 120859 | 119127 (99%) |  | 409 | 114890 | 109743 (96%) |

**Table S2. Summary of observed bacterial and fungal OTUs, Shannon, Simpson, ACE, Chao and coverage**

| Sample ID | **Bacteria** | | | | | |  | **Fungi** | | | | | |
| --- | --- | --- | --- | --- | --- | --- | --- | --- | --- | --- | --- | --- | --- |
|  | OTUs | Shannon | Simpson | Chao | ACE | Coverage |  | OTUs | Shannon | Simpson | Chao | ACE | Coverage |
| EBY-1 | 1077 | 6.90 | 0.98 | 1363.08 | 1350.63 | 1.00 |  | 197 | 3.82 | 0.85 | 221.33 | 235.39 | 1.00 |
| EBY-2 | 790 | 5.65 | 0.94 | 927.07 | 978.03 | 1.00 |  | 167 | 3.50 | 0.86 | 166.84 | 173.26 | 1.00 |
| EBY-3 | 859 | 4.84 | 0.92 | 1149.54 | 1230.30 | 1.00 |  | 228 | 4.08 | 0.83 | 256.28 | 262.56 | 1.00 |
| LS-1 | 663 | 5.47 | 0.94 | 868.15 | 906.06 | 1.00 |  | 204 | 4.24 | 0.87 | 223.77 | 224.24 | 1.00 |
| LS-2 | 603 | 4.78 | 0.91 | 741.98 | 764.26 | 1.00 |  | 135 | 3.14 | 0.94 | 153.40 | 158.60 | 1.00 |
| LS-3 | 1027 | 7.07 | 0.98 | 1266.05 | 1295.28 | 1.00 |  | 199 | 3.85 | 0.61 | 238.26 | 241.54 | 1.00 |
| XJY-1 | 942 | 4.41 | 0.93 | 1150.89 | 1195.64 | 1.00 |  | 106 | 2.53 | 0.77 | 123.60 | 120.72 | 1.00 |
| XJY-2 | 704 | 3.98 | 0.84 | 980.76 | 931.67 | 1.00 |  | 117 | 2.49 | 0.62 | 137.00 | 131.33 | 1.00 |
| XJY-3 | 942 | 4.37 | 0.75 | 1126.19 | 1182.24 | 1.00 |  | 81 | 2.70 | 0.59 | 88.86 | 89.47 | 1.00 |

**Table S3. Selection of bacterial genes involved in lignocellulose metabolism, nitrogen fixation and detoxification (with the PICRUSt2 software) as well as the enzyme-catalyzed reactions.**

| **Function** | **Number** | **KEGG gene description** | **Reaction (IUBMB)** |
| --- | --- | --- | --- |
| **Lignin** | EC[1.1.3.15] | (S)-2-hydroxy-acid oxidase | an (S)-2-hydroxy carboxylate + O_2_ = a 2-oxo carboxylate + H_2_O_2_ |
|  | EC[1.11.1.10] | Chloride peroxidase | RH + chloride + H_2_O_2_ = RCl + 2 H_2_O |
|  | EC[1.11.1.21] | Catalase peroxidase | (1) donor + H_2_O_2_ = oxidized donor + 2 H_2_O; (2) 2 H_2_O_2_ = O_2_ + 2 H_2_O |
|  | EC[1.11.1.5] | Cytochrome-c peroxidase | 2 ferrocytochrome c + H_2_O_2_ = 2 ferricytochrome c + 2 H_2_O |
|  | EC[1.11.1.6] | Catalase | 2 H_2_O_2_ = O_2_ + 2 H_2_O |
|  | EC[1.11.1.9] | Glutathione peroxidase | 2 glutathione + H_2_O_2_ = glutathione disulfide + 2 H_2_O |
|  | EC[1.14.13.82] | Vanillate monooxygenase | vanillate + O_2_ + NADH + H+ = 3,4-dihydroxybenzoate + NAD+ + H_2_O + formaldehyde |
| **Cellulose and hemicellulose** | EC[3.1.1.1] | Carboxylesterase | a carboxylic ester + H2O = an alcohol + a carboxylate |
|  | EC[3.2.1.21] | Beta-glucosidase | hydrolysis of terminal, non-reducing beta-D-glucosyl residues with release of beta D-glucose |
|  | EC[3.2.1.22] | Alpha-galactosidase | hydrolysis of terminal, non-reducing alpha-D-galactose residues in alpha-Dgalactosides, including galactose oligosaccharides, galactomannans and galactolipids |
|  | EC[3.2.1.23] | Beta-galactosidase | hydrolysis of terminal non-reducing beta-D-galactose residues in beta-D galactosides |
|  | EC[3.2.1.24] | Alpha-mannosidase | hydrolysis of terminal, non-reducing alpha-D-mannose residues in alpha-D mannosides |
|  | EC[3.2.1.25] | Beta-mannosidase | hydrolysis of terminal, non-reducing beta-D-mannose residues in beta-D mannosides |
|  | EC[3.2.1.31] | Beta-glucuronidase | a beta-D-glucuronoside + H_2_O = D-glucuronate + an alcohol |
|  | EC[3.2.1.37] | Xylan 1,4-beta-xylosidase | hydrolysis of (1->4)-beta-D-xylans, to remove successive D-xylose residues fromthe non-reducing termini |
|  | EC[3.2.1.4] | Cellulase | endohydrolysis of (1->4)-beta-D-glucosidic linkages in cellulose, lichenin andcereal beta-D-glucans |
|  | EC[3.2.1.51] | Alpha-L-fucosidase | an alpha-L-fucoside + H_2_O = L-fucose + an alcohol |
|  | EC[3.2.1.55] | Non-reducing end alpha-L-arabinofuranosidase | hydrolysis of terminal non-reducing alpha-L-arabinofuranoside residues in alpha-Larabinosides |
|  | EC[3.2.1.65] | Levanase | random hydrolysis of (2->6)-beta-D-fructofuranosidic linkages in (2->6)- beta-Dfructans (levans) containing more than 3 fructose units |
|  | EC[3.2.1.78] | Mannan endo-1,4-beta-mannosidase | random hydrolysis of (1->4)-beta-D-mannosidic linkages in mannans, galactomannans and glucomannans |
|  | EC[3.2.1.8] | Endo-1,4-beta-xylanase | endohydrolysis of (1->4)-beta-D-xylosidic linkages in xylans |
|  | EC[3.2.1.86] | 6-phospho-beta-glucosidase | 6-phospho-beta-D-glucosyl-(1->4)-D-glucose + H2O = D-glucose + D- glucose 6-phosphate |
|  | EC[3.2.1.89] | Arabinogalactan endo-beta-1,4-galactanase | the enzyme specifically hydrolyses (1->4)-beta-D-galactosidic linkages in type I arabinogalactans |
|  | EC[3.2.1.91] | Cellulose 1,4-beta-cellobiosidase | hydrolysis of (1->4)-beta-D-glucosidic linkages in cellulose and cellotetraose, releasing cellobiose from the non-reducing ends of the chains |
| **N2-fixation** | EC[1.18.6.1] | Nitrogenase iron protein NifH | 8 reduced ferredoxin + 8 H+ + N_2_ + 16 ATP + 16 H_2_O = 8 oxidized ferredoxin + H_2_+ 2 NH_3_ + 16 ADP + 16 phosphate |
| **Detoxification** | EC[3.1.1.1] | Carboxylesterase | a carboxylic ester + H_2_O = an alcohol + a carboxylate |

**Table S4. Predicted bacterial community functions in the *A. glabripennis* larval gut following feeding on different host tree species.**

| **Function** | **Number** | **EBY** | **LS** | **XJY** | ***P*-value** |
| --- | --- | --- | --- | --- | --- |
| **Lignin** | EC[1.1.3.15]Glycolate oxidase | 7189.8±3170.7a | 9476.8±4874.2a | 5501.5±2971.7a | 0.28 |
|  | EC[1.11.1.10]Chloride peroxidase | 10112.3±1197.3b | 7665.4±4035.7b | 24828.4±13405.3a | 8.31* |
|  | EC[1.11.1.21]Catalase peroxidase | 18568.7±3083.4b | 8537.1±3936.3b | 30521.2±14453.1a | 7.954* |
|  | EC[1.11.1.5]Cytochrome-c peroxidase | 13636.2±4103.6a | 9969.1±4286.4a | 30559.0±14340.0a | 1.503 |
|  | EC[1.11.1.6]Catalase | 33643.1±3735.8a | 25680.2±15205.6a | 34661.9±19782.2a | 0.114 |
|  | EC[1.11.1.9]Glutathione peroxidase | 60774.1±7805.0b | 28949.4±13020.1c | 96539.5±44577.2a | 11.547* |
|  | EC[1.14.13.82]Vanillate monooxygenase | 8037.4±1897.6a | 9058.0±4933.3a | 6853.7±4040.6a | 0.082 |
| **Cellulose and hemicellulose** | EC[3.1.1.1]Carboxylesterase | 14305.1±12035.5a | 5519.7±566.3a | 1560.0±609.7a | 3.038 |
|  | EC[3.2.1.21]Beta-glucosidase | 103617.4±52461.8a | 45708.5±20787.5b | 129245.4±85405.6a | 16.524* |
|  | EC[3.2.1.22]Alpha-galactosidase | 52679.1±35499.2a | 18836.5±10493.1a | 25925.2±16794.0a | 0.578 |
|  | EC[3.2.1.23]Beta-galactosidase | 104778.4±58028.1a | 46247.5±22563.8b | 106579.9±68097.3a | 12.415* |
|  | EC[3.2.1.24]Alpha-mannosidase | 29524.4±25387.7a | 1852.4±1045.0a | 9459.1±8498.2a | 0.854 |
|  | EC[3.2.1.25]Beta-mannosidase | 2697.0±916.8a | 2992.9±1143.3a | 11493.9±9527.7a | 0.806 |
|  | EC[3.2.1.31]Beta-glucuronidase | 639.1±319.9a | 1391.4±1009.8a | 653.5±246.2a | 0.47 |
|  | EC[3.2.1.37]Xylan 1,4-beta-xylosidase | 28158.0±21770.9a | 3717.3±2319.6a | 2998.7±1577.5a | 1.277 |
|  | EC[3.2.1.4]Cellulase | 26246.3±5165.5a | 17417.9±8011.9a | 26618.4±14958.0a | 0.259 |
|  | EC[3.2.1.51]Alpha-L-fucosidase | 57897.3±39592.8a | 20102.3±12243.1a | 73766.7±69906.0a | 0.345 |
|  | EC[3.2.1.55] Non-reducing end alpha-L-arabinofuranosidase | 40079.4±31553.0a | 5797.1±3045.1a | 13260.6±10787.4a | 0.87 |
|  | EC[3.2.1.65]Levanase | 734.0±481.3a | 1122.7±1033.7a | 878.0±738.5a | 0.063 |
|  | EC[3.2.1.78]  Mannan endo-1,4-beta-mannosidase | 2167.9±420.6b | 4772.1±2596.0b | 15784.8±15023.6a | 10.674* |
|  | EC[3.2.1.8]Endo-1,4-beta-xylanase | 4470.9±1631.7a | 2311.7±1121.6a | 11941.8±10067.6a | 0.728 |
|  | EC[3.2.1.86]6-phospho-beta-glucosidase | 247444.5±109230.7a | 59215.4±29437.0b | 155692.4±80664.2b | 16.377* |
|  | EC[3.2.1.89]  Arabinogalactan endo-beta-1,4-galactanase | 12465.8±9712.7a | 3307.4±2408.4a | 8517.5±7778.6a | 0.394 |
|  | EC[3.2.1.91]  Cellulose 1,4-beta-cellobiosidase | 161.8±92.2a | 23.0±22.5a | 27.5±22.6a | 1.96 |
| **N2-fixation** | EC[1.18.6.1]Nitrogenase | 1248.1±813.0a | 986.2±464.3a | 506.8±165.1a | 0.469 |
| **Detoxification** | EC[3.1.1.1]Carboxylesterase | 14305.1±12035.5a | 5519.7±566.3a | 1560.0±609.7a | 3.038 |

Note: Results followed by different letters are significantly different according to the HSD test (*0.01 < *p* ≤ 0.05).

**Table S5. Selection of fungal genes involved in lignocellulose metabolism and nitrogen fixation (with the PICRUSt2 software) as well as the enzyme-catalyzed reactions**

| **Function** | **Number** | **KEGG gene description** | **Reaction (IUBMB)** |
| --- | --- | --- | --- |
| **Lignin** | EC[1.10.3.2] | Laccase | 4 benzenediol + O(2) <=> 4 benzosemiquinone + 2 H(2)O |
|  | EC[1.1.3.15] | Glycolate oxidase | (S)-2-hydroxy acid + O(2) <=> 2-oxo acid + H(2)O(2) |
|  | EC[1.11.1.5] | Cytochrome-c peroxidase | 2 ferrocytochrome c + H_2_O_2_ = 2 ferricytochrome c + 2 H_2_O |
|  | EC[1.11.1.6] | Catalase | 2 H_2_O_2_ = O_2_ + 2 H_2_O |
|  | EC[1.11.1.9] | Glutathione peroxidase | 2 glutathione + H_2_O_2_ = glutathione disulfide + 2 H_2_O |
| **Cellulose and hemicellulose** | EC[3.1.1.1] | Carboxylesterase | a carboxylic ester + H_2_O = an alcohol + a carboxylate |
|  | EC[3.2.1.21] | Beta-glucosidase | hydrolysis of terminal, non-reducing beta-D-glucosyl residues with release of beta D-glucose |
|  | EC[3.2.1.22] | Alpha-galactosidase | hydrolysis of terminal, non-reducing alpha-D-galactose residues in alpha-Dgalactosides, including galactose oligosaccharides, galactomannans and galactolipids |
|  | EC[3.2.1.23] | Beta-galactosidase | hydrolysis of terminal non-reducing beta-D-galactose residues in beta-D galactosides |
|  | EC[3.2.1.24] | Alpha-mannosidase | hydrolysis of terminal, non-reducing alpha-D-mannose residues in alpha-D mannosides |
|  | EC[3.2.1.25] | Beta-mannosidase | hydrolysis of terminal, non-reducing beta-D-mannose residues in beta-D mannosides |
|  | EC[3.2.1.31] | Beta-glucuronidase | a beta-D-glucuronoside + H_2_O = D-glucuronate + an alcohol |
|  | EC[3.2.1.37] | Xylan 1,4-beta-xylosidase | hydrolysis of (1->4)-beta-D-xylans, to remove successive D-xylose residues fromthe non-reducing termini |
|  | EC[3.2.1.4] | Cellulase | endohydrolysis of (1->4)-beta-D-glucosidic linkages in cellulose, lichenin andcereal beta-D-glucans |
|  | EC[3.2.1.51] | Alpha-L-fucosidase | an alpha-L-fucoside + H_2_O = L-fucose + an alcohol |
|  | EC[3.2.1.55] | Non-reducing end alpha-L-arabinofuranosidase | hydrolysis of terminal non-reducing alpha-L-arabinofuranoside residues in alpha-Larabinosides |
|  | EC[3.2.1.65] | Levanase | random hydrolysis of (2->6)-beta-D-fructofuranosidic linkages in (2->6)- beta-Dfructans (levans) containing more than 3 fructose units |
|  | EC[3.2.1.78] | Mannan endo-1,4-beta-mannosidase | random hydrolysis of (1->4)-beta-D-mannosidic linkages in mannans, galactomannans and glucomannans |
|  | EC[3.2.1.8] | Endo-1,4-beta-xylanase | endohydrolysis of (1->4)-beta-D-xylosidic linkages in xylans |
|  | EC[3.2.1.39] | Glucan endo-1,3-beta-D-glucosidase | Hydrolysis of (1->3)-beta-D-glucosidic linkages in (1->3)-beta-D-glucans |
|  | EC[3.2.1.89] | Arabinogalactan endo-beta-1,4-galactanase | the enzyme specifically hydrolyses (1->4)-beta-D-galactosidic linkages in type I arabinogalactans |
|  | EC[3.2.1.91] | Cellulose 1,4-beta-cellobiosidase | hydrolysis of (1->4)-beta-D-glucosidic linkages in cellulose and cellotetraose, releasing cellobiose from the non-reducing ends of the chains |
| **Detoxification** | EC[3.1.1.7] | Acetylcholinesterase | Acetylcholine + H(2)O <=> choline + acetate |
|  | EC[3.1.1.1] | Carboxylesterase | a carboxylic ester + H_2_O = an alcohol + a carboxylate |

**Table S6. Predicted bacterial community functions in the *A. glabripennis* larval gut following feeding on different host tree species.**

| **Function** | **Number** | **EBY** | **LS** | **XJY** | **P-value** |
| --- | --- | --- | --- | --- | --- |
| **Lignin** | EC[1.1.3.15]Glycolate oxidase | 1487.0±770.8a | 1892.0±531.4a | 1656.7±468.1a | 0.113 |
|  | EC[1.10.3.2]Laccase | 11963.0±216.8a | 7139.7±800.8b | 4977.7±1747.2b | 10.256* |
|  | EC[1.11.1.5]Cytochrome-c peroxidase | 2922.0±2016.4a | 3412.3±1754.9a | 3345.7±1465.3a | 0.023 |
|  | EC[1.11.1.6]Catalase | 5168.7±2598.4a | 7381.7±2391.0a | 8782.0±3026.6a | 0.46 |
|  | EC[1.11.1.9]Glutathione peroxidase | 1742.7±1120.6a | 3144.7±1563.5a | 3209.0±1536.5a | 0.34 |
| **Cellulose and hemicellulose** | EC[3.1.1.1]Carboxylesterase | 10645.0±5313.6a | 9433.3±4229.0a | 3743.3±1624.1a | 3.836 |
|  | EC[3.2.1.21]Beta-glucosidase | 19830.3±9807.0a | 24160.7±4576.0a | 28899.7±8735.1a | 0.319 |
|  | EC[3.2.1.22]Alpha-galactosidase | 3351.0±1634.9a | 3679.7±804.9a | 6048.7±1843.7a | 0.967 |
|  | EC[3.2.1.23]Beta-galactosidase | 2972.7±1397.4a | 4104.3±1335.1a | 7800.7±2773.0a | 1.674 |
|  | EC[3.2.1.24]Alpha-mannosidase | 1746.0±1118.2a | 2463.0±1072.3a | 2438.7±929.4a | 0.152 |
|  | EC[3.2.1.25]Beta-mannosidase | 3315.0±1639.9a | 4155.3±1081.1a | 5575.0±1851.7a | 0.537 |
|  | EC[3.2.1.31]Beta-glucuronidase | 1168.7±731.8a | 1908.3±496.7a | 3053.0±1150.7a | 1.284 |
|  | EC[3.2.1.37]Xylan 1,4-beta-xylosidase | 153.3±73.3a | 948.0±530.7a | 3622.3±1551.8a | 3.676 |
|  | EC[3.2.1.39]  Glucan endo-1,3-beta-D-glucosidase | 356.0±298.0a | 898.0±79.2a | 2572.3±1046.3a | 3.366 |
|  | EC[3.2.1.4]Cellulase | 428.3±221.4b | 2326.7±1186.2a | 8009.3±3053.5a | 11.331* |
|  | EC[3.2.1.51]Alpha-L-fucosidase | 706.0±346.2a | 1139.0±190.7a | 2145.7±890.3a | 1.725 |
|  | EC[3.2.1.55]Non-reducing end alpha-L-arabinofuranosidase | 969.0±455.0a | 1125.7±151.2a | 2778.7±1370.3a | 1.431 |
|  | EC[3.2.1.65]Levanase | 8.0±6.1b | 2.7±0.3b | 379.0±359.2a | 17.082* |
|  | EC[3.2.1.78]Mannan endo-1,4-beta-mannosidase | 130.3±75.8a | 1933.0±1520.6a | 2252.3±1299.7a | 4.98 |
|  | EC[3.2.1.8]Endo-1,4-beta-xylanase | 238.3±162.7b | 693.3±166.4b | 3234.0±1359.8a | 14.108* |
|  | EC[3.2.1.89]  Arabinogalactan endo-beta-1,4-galactanase | 1110.3±534.5a | 1123.7±150.4a | 1642.0±468.6a | 0.522 |
|  | EC[3.2.1.91]  Cellulose 1,4-beta-cellobiosidase | 48.3±17.4b | 240.3±132.3b | 1379.3±555.2a | 14.763* |
| **Detoxification** | EC[3.1.1.1]Carboxylesterase | 10645.0±5313.6a | 9433.3±4229.0a | 3743.3±1624.1a | 3.836 |
|  | EC[3.1.1.7]Acetylcholinesterase | 4727.7±2561.0a | 3774.7±223.5a | 4941.0±1396.0a | 0.135 |

Note: Results followed by different letters are significantly different according to the HSD test (*0.01 < *p* ≤ 0.05).
